# Racial/Ethnic Disparities in Crime Exposure and Associations With Middle-Aged and Older Americans’ Mental Health

**DOI:** 10.1093/geroni/igaf122.649

**Published:** 2025-12-31

**Authors:** Junjie He, Zhuoer Lin, Yan Zhang, Yang Wang, Xi Chen

**Affiliations:** Yale University, State College, Pennsylvania, United States; University of Illinois Chicago, Chicago, Illinois, United States; Texas State University, Greenville, North Carolina, United States; University of Wisconsin–Madison, Madison, Wisconsin, United States; Yale University, New Haven, Connecticut, United States

## Abstract

While the association between crime and mental health is documented, its racial and ethnic differences and interactions with crime types on late-life mental health remain unclear. This study examined the impact of crime exposure on depressive symptoms, focusing on racial and ethnic disparities and main crime types. We linked the Health and Retirement Study (HRS), a nationally representative longitudinal data, with crime data from the Uniform Crime Reporting (UCR) system. The final analytic sample included 27,899 Americans aged 50 and older. Crime exposure was measured by local crime rates at the county-year level and depressive symptoms were measured by the CES-D scale. Employing models with individual fixed-effects and adjusting for comprehensive covariates to mitigate potential biases, we found that a one-standard-deviation increase in local crime rates was associated with a 1.03% increase in the likelihood of experiencing depressive symptoms. Compared to non-Hispanic Whites, non-Hispanic Blacks and Hispanics were both exposed to higher crime levels and exhibited more depressive symptoms. Moreover, the negative effects of crime were stronger for non-Hispanic Blacks and Hispanics than for Whites, with significantly larger effects of both violent and property crimes for non-Hispanic Blacks, and larger effects of property crimes for Hispanics. These patterns held across various robustness checks. Our findings suggest that persistent racial and ethnic disparities in late-life mental health may be driven by structural racial and ethnic inequities, in particular through differential exposure to crime and the amplified impact of crime, highlighting the importance of enhancing community safety to address health disparities.

